# Development of an interactive curriculum and trainee-specific preparedness plan for emergency medicine residents

**DOI:** 10.1186/s12245-020-00295-9

**Published:** 2020-07-14

**Authors:** Ayanna D. Walker, Nicholas Fusco, Joshua Tsau, Latha Ganti

**Affiliations:** 1 UCF/HCA Emergency Medicine Residency Program of Greater Orlando, 700 W. Oak St., Kissimmee, FL 33471 USA; 2 Envision Physician Services, 700 W. Oak St., Kissimmee, FL 33471 USA

**Keywords:** Emergency Medicine Residency Curriculum, Disaster training, Mass casualty incident (MCI)

## Abstract

**Objectives:**

To create an interactive mass casualty incident (MCI) curriculum for emergency medicine residents and to integrate them into the hospital disaster response, thereby creating a “trainee-specific emergency preparedness plan.”

**Methods:**

We created an interactive MCI curriculum and “trainee-specific emergency preparedness plan” for emergency medicine residents. The curriculum consisted of lectures, a small focus group, a triage activity, and the designation of a resident disaster champion to collaborate with hospital leadership to implement a “trainee-specific emergency preparedness plan” for the upcoming hospital disaster drill.

**Results:**

Residents gave positive feedback on the new curriculum and retained information from the education. All resident teams accurately triaged at least 78% of the disaster scenarios. The residents also created a “trainee-specific emergency preparedness plan” for the upcoming hospital disaster drill, utilizing principles they learned from their MCI lessons. By allowing the residents to have an active role in the design and implementation of the new resident integrated disaster management plan, there was a general consensus of increased interest and retention of what was learned, as well as an increased comfort level in participating in MCI scenarios. Residents did not feel cursory to the planning; they became a part of the planning and felt more involved. Through this exercise, residents were able to give feedback to the hospital leadership that further shaped the disaster response plan. We also found that integration of the emergency medicine residents into the hospital response doubled the amount of active physicians available.

**Conclusion:**

An interactive-based MCI curriculum is more engaging and may foster more retention than the traditional lecture approach. Resident involvement in the hospital disaster response is paramount as more hospitals are becoming teaching hospitals and mass casualty incidents are inevitable.

## Introduction

Per the World Health Organization (WHO), “a mass casualty incident is defined as an event which generates more patients at one time than locally available resources can manage using routine procedures. It requires exceptional emergency arrangements and additional or extraordinary assistance” [1]. Mass casualty incidents (MCI) have been an unfortunately increasing occurrence in the USA over the last decade. Events like the Sandy Hook shooting in California, Boston Marathon Bombing in Massachusetts, Pulse Nightclub shooting in Orlando Florida, Las Vegas shooting, and the shootings in El Paso Texas and Dayton Ohio have been some of the worst mass casualty incidents in US history. The mitigating feature of these horrible situations has been the readiness and training of the emergency response team. Given the accelerating occurrence of MCIs, the need for a formalized curriculum for the Emergency Medicine resident is becoming increasingly necessary to provide the best care for the patients affected by these abhorrent occurrences. Residency provides a unique opportunity to provide focused training for MCIs, prior to being placed in a real-time MCI scenario.

Currently, there is no standard curriculum in emergency medicine for mass casualty incidents. In fact, many physicians may not ever receive disaster education and training. One study showed an overall low confidence level in managing MCIs among residents [2]. In addition, 68.5% of 183 Emergency Medicine Residency programs reported that lectures are the most common method of instruction [3] even though previous studies have shown that residents prefer more interactive and simulation-based learning environment. One can surmise that the current training that is provided is not adequate for preparing the emergency medicine resident for real-world scenarios.

Many hospitals are required to participate in disaster preparedness events; however, there is scarcity of literature that illustrates resident involvement in these simulations.

Inevitably, with the exponential rise in residencies, there will be questions surrounding residency involvement in the disaster response and how they should be or if they should be integrated into the hospital response. Questions will come up such as follows: “should they be included in the surge staffing,” “how would this determination be made,” “how would they be contacted,” and “what would be their role?” As residents make up a significant portion of the hospital staff, providing a trainee-specific emergency preparedness plan will likely enable residents to better manage these events, and it will provide a strong resource for the hospital during these unfortunate occurrences.

## Methods

Our goal was to implement an interactive curriculum and “trainee-specific emergency preparedness plan” [4]. MCI education was the initial objective of the developing curriculum. Topics of the NIMS (National Incident Management System) and triage tools were discussed to lay the initial foundation of MCI management. An exercise was then utilized to assess triage skills using the START (simple triage and rapid treatment) and JumpSTART algorithms [5]. For this exercise, a group of 14 residents were split into five teams and asked to rapidly triage fourteen volunteer patients with predetermined vital signs and injuries. The PGY 1 residents and visiting medical students served as the volunteer patients.

The next stage of our developing MCI curriculum involved a small focus group of Post Graduate Year III (PGYIII) residents after reviewing EM Foundations III [6]. EM Foundations III is a course within Foundations of Emergency Medicine, a national flipped classroom and case-based curriculum designed for emergency medicine residents. During this discussion, the residents were required to locate the hospital’s disaster plan, review it, and then discuss its implementation should an MCI occur. After the foundations activity, the residents appointed a “Disaster Champion,” whose roles included collaborating with emergency department leadership in the development of a trainee-specific emergency preparedness plan with the purpose of integrating residents into the current disaster management plan, as well as to educate the residents on the newly created trainee-specific plan, observe the residents during the hospital MCI drill, coordinate a residency wide debrief, and communicate their observations and suggestions for improvement to the hospital’s emergency management committee.

## Results and discussion

**Fig. 1:**
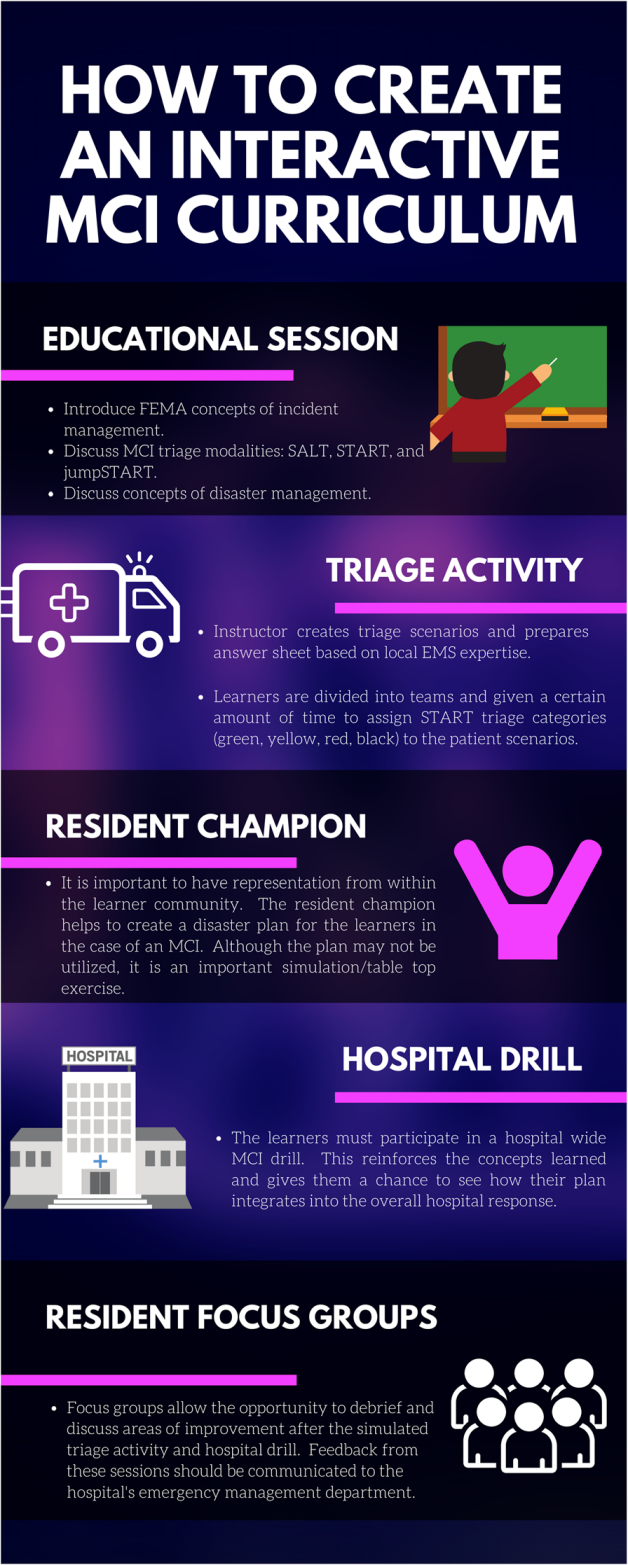
Interactive MCI curriculum

The year prior to implementation of the new interactive MCI curriculum, there was only a 30-min disaster lecture presented during one of the residents’ weekly conferences. There was no positive feedback given by the residents and when retention was informally tested, residents did not recall key components of the disaster response. In contrast, after the institution of the interactive MCI curriculum [Fig. 1], residents performed well during a mock triage activity, demonstrating retention of key disaster principles. Using the START and jumpSTART triage tools, all resident teams accurately triaged at least 78% of the disaster scenarios correctly, according to the predefined triage category assigned by the local EMS medical director.

As a part of the interactive curriculum, PGYIII residents were to review the hospital’s disaster plan in a small group. During the review, we found that there was no inclusion of the residents within the plan. This posed a problem because residents were aware of an upcoming hospital wide disaster drill and were inquiring about their roles and responsibilities for the drill should a real MCI occur. The fact that there was no plan for residents actually became an integral part of the interactive curriculum. This inspired them to create a plan that integrated residents, utilizing fundamental principles they learned in their MCI lessons. Specifically, the residents determined their own roles and responsibilities for the upcoming hospital MCI Drill.

Prior to implementation of our MCI curriculum, the residents were not involved in planning or implementation of disaster drills and management. By allowing the residents to have an active role in both the design and implementation of the new resident integrated disaster management plan, there was a general consensus of increased interest and retention of what was learned but also increased comfort level in management and participating in MCI scenarios. Residents became a part of the planning and felt more involved.

When it came time for the hospital MCI drill, which occurred 2 weeks after the interactive MCI curriculum, the resident disaster champion was key in communicating roles and responsibilities to residents and attendings within the emergency department. The plan divided the residents and attendings into treatment zones based on PGY year, with each treatment zone having a PGY III as the lead treatment officer. PGY I residents were assigned to the green treatment zone, PGY II residents to the yellow treatment zone, and PGY III residents to the red treatment zone. Over 121 volunteer patients were triaged by 2 PGY III residents and 2 attendings. Observations made by the disaster champion and a debriefing session with the residents brought up the need for the following: additional MCI-specific treatment supplies in each zone, a better communication method for each zone, consistent documentation, a clear disposition plan, and the need to integrate other residencies. This feedback was given to the hospital emergency management committee. Through this exercise, we also found that integration of the emergency medicine residents into the hospital response doubled the amount of active physicians available during the MCI.

## Limitations

A major challenge was integrating the residents into the hospital emergency management committee so close to the actual date of the MCI drill. We thought it was important to include the residents in the exercise however, because it would help them to become familiar with the hospital notification of an MCI, understand the command structure, understand their roles, and become familiar with tools used during a hospital wide disaster, such as documentation tools, identifying vests, and triage tags. A second limitation was that there were 7 residents that participated in the prior curriculum that did not take part in this new curriculum. Therefore, no comparison was able to be made from their perspective. Finally, the proximity of the discussion of MCI principles with the mock triage activity presents a limitation as it relates to knowledge retention. It would be helpful to repeat the mock triage activity months after the MCI discussion to measure change over time.

## Conclusions

With the rise in occurrences of nationwide mass casualty incidences, it is paramount that physicians receive disaster training. Utilizing an interactive curriculum to educate residents about MCIs better prepares them to respond to these and other events where local and regional resources are overwhelmed. In addition to disaster training in residency, hospitals will need to incorporate residents into their emergency management plan. The integration of emergency medicine residents into the emergency management plan is even more necessary as more hospitals are becoming teaching hospitals and have a significant amount of physicians that are trainees.

## Data Availability

N/A

## Abbreviations

ED: Emergency department
WHO: World Health Organization
MCI: Mass casualty incident
NIMS: National Incident Management System
PGY III: Post Graduate Year III
